# Comparison of Genetic and Self-Identified Ancestry in Modeling Intracerebral Hemorrhage Risk

**DOI:** 10.3389/fneur.2018.00514

**Published:** 2018-07-06

**Authors:** Sandro Marini, Umme K. Lena, Katherine M. Crawford, Charles J. Moomaw, Fernando D. Testai, Steven J. Kittner, Michael L. James, Daniel Woo, Carl D. Langefeld, Jonathan Rosand, Christopher D. Anderson

**Affiliations:** ^1^Center for Genomic Medicine, Massachusetts General Hospital, Boston, MA, United States; ^2^Medical and Population Genetics, Broad Institute, Cambridge, MA, United States; ^3^Department of Neurology and Rehabilitation Medicine, University of Cincinnati College of Medicine, Cincinnati, OH, United States; ^4^Department of Neurology and Rehabilitation, University of Illinois College of Medicine, Chicago, IL, United States; ^5^Department of Neurology, Baltimore Veterans Administration Medical Center and University of Maryland School of Medicine, Baltimore, MD, United States; ^6^Departments of Anesthesiology and Neurology, Brain Injury Translational Research Center, Duke University, Durham, NC, United States; ^7^Center for Public Health Genomics and Department of Biostatistical Sciences, Wake Forest University, Winston-Salem, NC, United States; ^8^J. Philip Kistler Stroke Research Center, Massachusetts General Hospital, Boston, MA, United States

**Keywords:** genetics population, precision medicine, vascular diseases, risk factors, genetics, intracranial hemorrhage, race and ethnicity

## Abstract

**Background:** We sought to determine whether a small pool of ancestry-informative DNA markers (AIMs) improves modeling of intracerebral hemorrhage (ICH) risk in heterogeneous populations, compared with self-identified race/ethnicity (SIRE) alone.

**Methods:** We genotyped 15 preselected AIMs to perform principal component (PC) analysis in the ERICH study (a multi-center case-control study of ICH in whites, blacks, and Hispanics). We used multivariate logistic regression and tests for independent samples to compare associations for genetic ancestry and SIRE with ICH-associated vascular risk factors (VRFs). We then compared the performance of models for ICH risk that included AIMs and SIRE alone.

**Results:** Among 4,935 subjects, 34.7% were non-Hispanic black, 35.1% non-Hispanic white, and 30.2% Hispanic by SIRE. In stratified analysis of these SIRE groups, AIM-defined ancestry was strongly associated with seven of the eight VRFs analyzed (*p* < 0.001). Within each SIRE group, regression of AIM-derived PCs against VRFs confirmed independent associations of AIMs across at least two race/ethnic groups for seven VRFs. Akaike information criterion (AIC) (6,294 vs. 6,286) and likelihood ratio test (*p* < 0.001) showed that genetic ancestry defined by AIMs achieved a better ICH risk modeling compared to SIRE alone.

**Conclusion:** Genetically-defined ancestry provides valuable risk exposure information that is not captured by SIRE alone. Particularly among Hispanics and blacks, inclusion of AIMs adds value over self-reported ancestry in controlling for genetic and environmental exposures that influence risk of ICH. While differences are small, this modeling approach may be superior in highly heterogeneous clinical poulations. Additional studies across other ancestries and risk exposures are needed to confirm and extend these findings.

## Introduction

Vascular risk factors (VRFs) are often stratified by race and ethnicity. Hypertension has well-documented racial differences in prevalence, with African Americans more frequently hypertensive than whites(1–3). These disparities in hypertension impact both disease risk [the population attributable risk varies from 80.3 to 97.6% (4, 5)] and outcomes (6). Variability in risk exposure by race/ethnicity extends to other VRFs as well. Whites tend to have more unfavorable lipid profiles compared to blacks (7), and diabetes prevalence varies by race/ethnicity from 7.1% in whites to more than 10% in Hispanics and blacks (8). These ethnic and racial differences extend to the epidemiology of intracerebral hemorrhage (ICH), with increased risk in blacks and Hispanics compared with non-Hispanic whites (9, 10). As such, approaches that maximize the capture of racial and ethnic exposures in modeling of disease risk may improve the precision of our risk assessments.

In genome-wide association studies (GWAS), genetic polymorphisms whose allele frequencies vary markedly across different racial and ethnic groups [i.e., ancestry informative markers (AIMs)] are typically used to control for biases resulting from population stratification in their analyses (11). However, outside of GWAS, differences in risk factor prevalence among populations are usually addressed by using self-identified race and ethnicity (SIRE) information. Although SIRE does capture common cultural and social distinctions, it does not capture the full spectrum of genetic and ancestral diversity, nor does it adequately control for varying exposures within ancestral groups (12). Strengths and weaknesses of both approaches have been described elsewhere (13–16), but the use of AIMs to control for ancestry-related exposures remains rare outside of genetic association studies. As such, the potential benefits of leveraging AIMs data to control for racial and ethnic risk factors in epidemiologic studies has not been well-studied (17, 18).

We hypothesized that AIMs can provide value in capturing risk exposures that vary within and between ancestral populations only partially captured by SIRE (19). In testing this hypothesis, we utilized data from the Ethnic/Racial Variations of Intracerebral Hemorrhage (ERICH) study, which contains information on self-reported race and ethnicity from a standardized questionnaire, as well as AIMs data from a small pool of highly-informative variants in ICH cases and stroke-free controls.

## Materials and methods

### Source sample and clinical variables

The ERICH study is a prospective, multicenter, case-control study of ICH, designed to identify the genetic and epidemiologic factors that affect risk of ICH in a multi-ethnic population. As previously described (20), ERICH recruited approximately equal numbers of self-identified non-Hispanic white (whites), non-Hispanic black (blacks), and Hispanic patients aged ≥18 years with spontaneous ICH (defined as the sudden onset of severe headache, altered level of consciousness, or focal neurologic deficit associated with a focal collection of blood within the brain parenchyma, seen on neuroimaging) across 19 clinical recruitment centers. Cases of peripartum and anticoagulant-associated ICH were included, whereas ICH due to malignancy-associated coagulopathy, dural venous sinus thrombosis, vascular malformations, aneurysms, tumors, or hemorrhagic conversion of a recent ischemic stroke did not qualify as study cases. Controls were identified through random digit dialing to match cases by age (±5 years), sex, race/ethnicity, and geographic area. IRB approval was obtained at all participating centers and informed consent was obtained from all cases or their legally authorized representative.

All cases (or designated proxies) and controls underwent a standardized data collection protocol based on personal interviews. Among the items on baseline interview we focused on variables with established associations with cerebrovascular disease in order to examine their stratification among and within each included race/ethnicity (21). Namely, we analyzed self-reported history of hypertension, diabetes mellitus (DM), hyperlipidemia, coronary artery disease (CAD), carotid artery disease, nephropathy/kidney disease, ischemic stroke, and atrial fibrillation. This approach allowed us to compare cases and controls (that do not have chart abstraction) without over-representing the risk factors in cases. Additionally, to gage whether the prevalence of self-reported hypertension was erroneous, and to exclude systematic reporting bias, we collected also blood pressure readings [treated as dichotomous variable according to the new American College of Cardiology and American Heart Association guidelines (22)]. As in other studies (23), SIRE was classified as: non-Hispanic whites, non-Hispanic blacks, and Hispanics (non-African ancestral Hispanics). Genotype data used to create the ancestral PCs and admixture tables for this analysis are available for download from the International Stroke Genetics Consortium's Cerebrovascular Disease Knowledge Portal (cerebrovascularportal.org) (24). Software tools employed in our analyses were not modified or customized for our use.

### Selection and genotyping of AIMS

For each of the three possible pairs of ancestral populations, we identified markers in which the difference in allele frequency (δ) was at least 0.5 between any two of the recruited ancestral populations. This allele frequency difference was chosen in order to maximize the resolution of ancestral populations with the minimum genotyping requirement. With these restrictions, we identified 15 AIMs that were adequately distributed across the genome (Supplement Table I). Genotyping of ERICH samples was performed using OpenArray TaqMan assay at the University of Miami. The 15 AIMs used in this study were bi-allelic single nucleotide polymorphisms (SNPs) that were selected based on information content for ancestry in the ancestral populations studied. Samples with < 90% call rates, or SNPs with call rates < 95% or deviation from Hardy-Weinberg equilibrium (at *P*-value < 2.5 × 10^−4^ for cases and at *P*-value < 2.5 × 10^−2^ for controls) were excluded.

### Statistical analysis

To test for association with VRF across SIRE groups, we computed chi-square tests of independence for the baseline characteristics and Mann-Whitney U tests for the ancestry principal components (PC). Similarly, we computed the Mann-Whitney U tests to test for association between the SIRE groups and the ancestral PC. We used logistic regression (adjusted for age and sex) to assess the role of PCs in predicting each VRF, within each self-identified population. To perform PC analysis, we used PLINK v1.9 variance-standardized relationship matrix dimension reduction (25). We combined the results across the SIRE groups using a meta-analysis approach. Specifically, within each SIRE group, we performed backward stepwise regressions (likelihood ratio method) adjusted for age and sex to select the best PC predicting each VRF. We then carried on the absolute values obtained in the inverse variant weighted random effects meta-analysis. We assessed heterogeneity between studies using the Cochran's Q and I^2^ statistics [derSimonian-Laird estimate (26)].

As a confirmatory approach, we used the software program ADMIXTURE (27) to compute estimates of the three population ancestral proportions (one for each of the three ancestral populations inferred given the PC plot and the Cross-validation errors) using the same autosomal SNP genotypes. We used the three ancestry proportions as the independent variable, similarly to the PC in the previous approach, to test for associations as outlined above.

Finally, VRFs, age and SIRE with and without PCs 1–3 were modeled as predictors of ICH, and Akaike information criterion (AIC) and likelihood ratio (28) were used to compare these regression models. Additional models adjusting for centers of recruitments were performed.

As an additional approach to compare the difference between the different models, we also used net reclassification improvement (NRI). We used the cutoff of 30% risk of hemorrhage. This corresponds to a score of >4 points of the ATRIA Bleeding Risk Score, that is the threshold commonly used in clinical settings when assessing eligibility for anticoagulation treatment (29, 30) We performed NRI analyses using the Predictable package in R (31). We used R software, version 3.4.1 (The R Foundation for Statistical Computing) and the statistical package SPSS v. 21, 2012 (www.spss.com). All significance tests were 2-tailed.

## Results

The 4,935 subjects with genotype data were 43% female and had a median age of 61 years [interquartile range (IQR): 52–72]. The SIRE groups were relatively balanced between 34.7% non-Hispanic blacks, 35.1% non-Hispanic whites, and 30.2% Hispanics. There were 2,793 ICH cases (median age 60, IQR 51–73; male 59.2%), with the percentage of cases comparable within each SIRE (33.8% for blacks, 32.7% for whites, and 33.5% for Hispanics) (Table 1). Self-reported history of hypertension and blood pressure readings were highly correlated (Spearman's rho 0.138, *p* < 0.001).

**Table 1:** Sample demographic and clinical characteristics (*n* = 4935).

	**Whites**	**Blacks**	**Hispanics**	*p*-value^*^
Male sex, *n* (%)	975 (56.2)	957 (55.9)	879 (59.0)	0.163
ICH, *n* (%)	913 (52.7)	943 (55.1)	937 (62.9)	<0.001
Age, median (IQR)	69 (59–79)	57 (50–65)	58 (49–68)	
Hypertension, *n* (%)	1068 (61.9)	1269 (74.8)	969 (65.8)	<0.001
Diabetes, *n* (%)	318 (18.4)	386 (22.7)	436 (29.4)	<0.001
Hypercholesterolemia, *n* (%)	840 (49.9)	614 (37.0)	588 (41.3)	<0.001
History of ischemic stroke, *n* (%)	94 (5.4)	97 (5.8%)	99 (6.7)	0.312
History of carotid artery disease, *n* (%)	54 (3.1)	17 (1.0)	18 (1.2)	<0.001
History of Kidney Damage/Nephropathy, *n* (%)	84 (8.9)	127 (7.5)	104 (7.0)	0.003
History of atrial fibrillation, *n* (%)	214 (12.4)	64 (3.8)	66 (4.4)	<0.001
History of History of coronary artery disease, *n* (%)	245 (14.2)	120 (7.1)	127 (8.6)	<0.001
Ever-smoker, *n* (%)	869/1729 (50.3)	883/1706 (51.8)	638/1485 (43.0)	<0.001
Alcohol use (more than once a day), *n* (%)	64/152 (42.1)	73/212 (34.4)	55/132 (41.7)	0.239

* *Chi-square test between to test for not equality between groups*.

### Genetic and self-identified ancestry cluster populations

**Figure 1:**
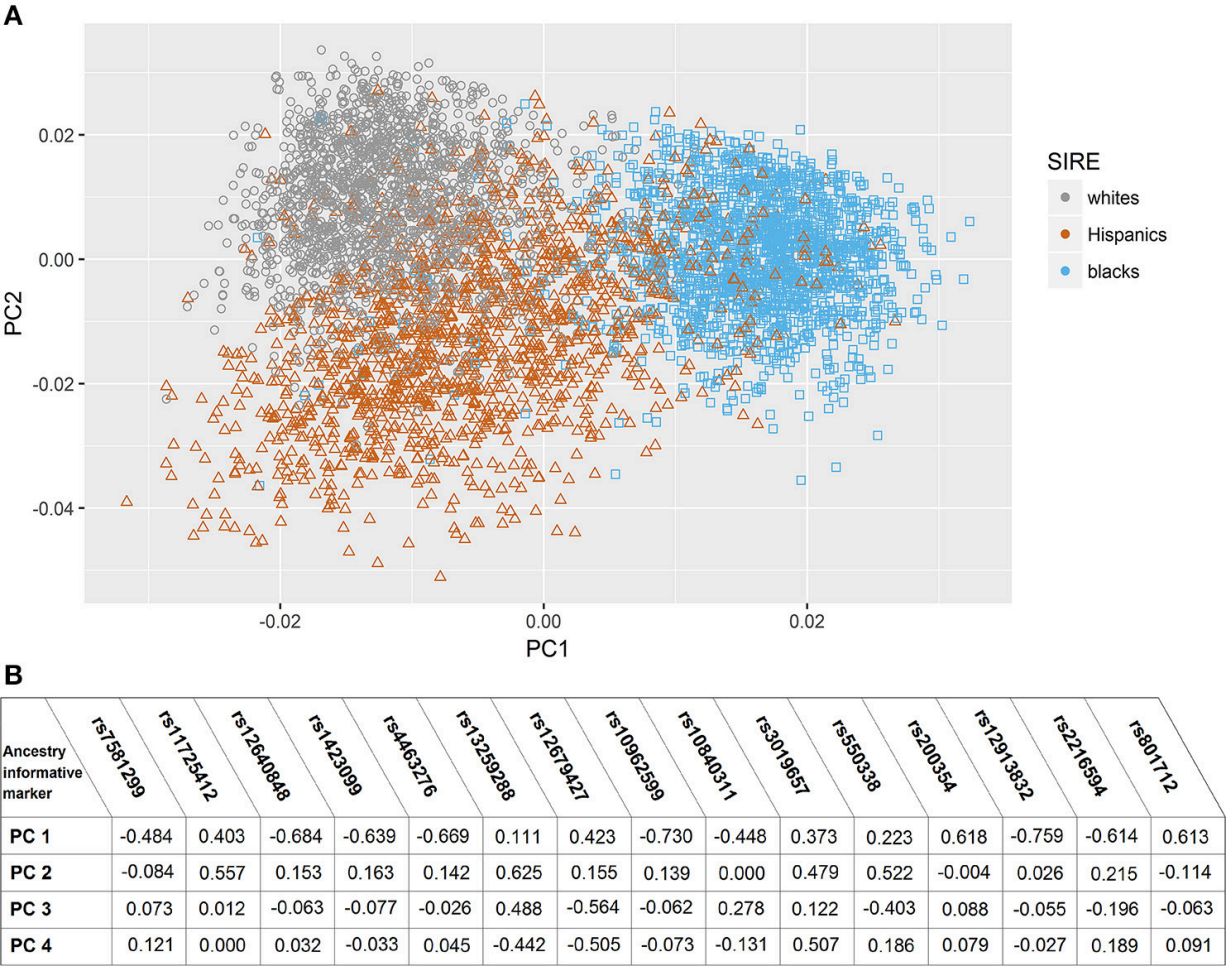
Principal component analysis based on ancestry informative markers. **(A)** Scatter plot of subjects in Principal Component (PC) space, grouped by their self-identified race/ethnicity. **(B)** Correlation coefficients between eigenvactors (top 4) and the Ancestry informative markers selected genotypes.

In the assessment of genetic admixture between groups defined by SIRE, there was incomplete overlap between ancestry-based clusters and SIRE (Figure 1) (Supplement Figure I). reflecting considerable admixture between self-identified groups. The result of the PC analysis indicated two primary PCs (Supplement Figure II). Unsupervised reclassification of subjects based on their position in AIM-derived PCs space (Supplement Figure I) showed that 4.1% of whites, 2.6% of blacks, and 2.9% of Hispanics by SIRE would be reclassified to a different racial/ethnic population based on the AIMs-based PC clusters assignment alone (Supplement Table II). Similar results were obtained with the ancestry fractions (AF) obtained from ADMIXTURE (Supplement Figure III). Correlation between AF and PCs are shown in Supplement Table III.

### Frequency of vascular risk factors in groups defined by self-described and genetically determined ancestry

Self-identified race/ethnicity (SIRE) and genetic ancestry determined by AIM-based PCs were both tested for association with reported VRF diagnoses. Prevalence of hypertension, hypercholesterolemia, diabetes, CAD, atrial fibrillation, history of nephropathy, and carotid disease differed across SIRE, while AIM-derived PCs were associated with each of these VRFs (all *p* < 0.001) (Table 2, Supplement Figure IV). For history of ischemic stroke, there was no association with either PCs or SIRE. When stratified by SIRE, AIMs-based PC remained significantly associated with multiple VRFs (*p* < 0.001) (Table 3), indicating that genetic ancestry yielded independent information from SIRE relative to these VRFs. For each VRF except hypercholesterolemia, AIMs-based PC were significantly different between affected and unaffected individuals within one or multiple SIRE groups. For example, self-reported blacks demonstrated fine-scale ancestral differences based on hypertension diagnosis. Similarly, a history of CAD was associated with residual genetic ancestry across all three populations studied. These PC-based results were comparable to those obtained when comparing ancestral proportions from ADMIXTURE (see section Methods) in the entire ERICH sample (Supplement Table IV) and within the SIRE groups (Supplement Table V).

**Table 2:** Distribution of vascular risk factors across genetic ancestry (Principal Component 1) among affected and non-affected individuals.

	**Yes**	**No**	***p*-value**
**HYPERTENSION**			
PC1 Median (SD)	−0.0052 (0.0258)	0.0011 (0.0260)	<0.001
**HYPERCHOLESTEROLEMIA**			
PC1 Median (SD)	0.0002 (0.0265)	−0.0054 (0.0254)	<0.001
**DIABETES**			
PC1 Median (SD)	−0.0060 (0.0240)	−0.0025 (0.0266)	<0.001
**CORONARY ARTERY DISEASE**			
PC1 Median (SD)	0.0072 (0.0255)	−0.0045 (0.0260)	<0.001
**ATRIAL FIBRILLATION**			
PC1 Median (SD)	0.0117 (0.02293)	−0.0045 (0.0261)	<0.001
**HISTORY OF ISCHEMIC STROKE**			
PC1 Median (SD)	−0.0053 (0.0250)	−0.0033 (0.0263)	0.071
**HISTORY OF NEPHROPATHY**			
PC1 Median (SD)	−0.0078 (0.0228)	−0.0031 (0.0264)	0.001
**HISTORY OF CAROTID DISEASE**			
PC1 Median (SD)	0.0130 (0.0261)	−0.0039 (0.0262)	<0.001

*Independent-Samples Mann-Whitney U test. PC, principal component; SD, standard deviation*.

**Table 3:** PCs differentiate those affected from those unaffected by VRFs, within each self-identified race/ethnicity group.

	**PC**	**Yes**	**No**	**Standardized test statistic**	***p*-value**
**HYPERTENSION**					
Whites	3	866.27	818.40	1.956	0.050
Blacks	1	816.15	897.36	−2.980	0.003
Hispanics	1	718.51	740.38	−0.943	0.346
**HYPERCHOLESTEROLEMIA**					
Whites	2	848.55	808.45	1.706	0.088
Blacks	1	824.11	812.84	0.466	0.641
Hispanics	1	721.77	687.11	1.580	0.114
**DIABETES**					
Whites	1	897.05	840.62	1.836	0.066
Blacks	1	804.23	849.93	−1.622	0.105
Hispanics	1	680.89	753.56	−3.005	0.003
**HISTORY OF CORONARY ARTERY DISEASE**					
Whites	3	907.63	835.81	2.111	0.035
Blacks	3	956.11	826.79	2.817	0.005
Hispanics	1	719.64	827.87	2.760	0.006
**HISTORY OF ATRIAL FIBRILLATION**					
Whites	1	807.90	853.10	−1.253	0.210
Blacks	1	979.25	828.91	2.412	0.016
Hispanics	3	738.26	619.62	−2.210	0.027
**HISTORY OF ISCHEMIC STROKE**					
Whites	3	855.89	748.28	−2.056	0.040
Blacks	4	731.48	835.52	−2.067	0.039
Hispanics	11	812.34	723.06	2.019	0.043
**HISTORY OF NEPHROPATHY**					
Whites	6	733.98	854.32	−2.171	0.030
Blacks	1	822.41	838.73	−0.364	0.716
Hispanics	2	636.71	736.09	−2.321	0.020
**HISTORY OF CAROTID DISEASE**					
Whites	10	677.56	854.12	−2.607	0.009
Blacks	1	840.24	767.15	−0.619	0.536
Hispanics	10	733.97	533.33	−2.004	0.045

*Median rank is reported for each category (yes/no) for each of the vascular risk factor*.

*p-value for independent-Samples Mann-Whitney U test. MR, mean rank; PC, principal component*.

**Figure 2:**
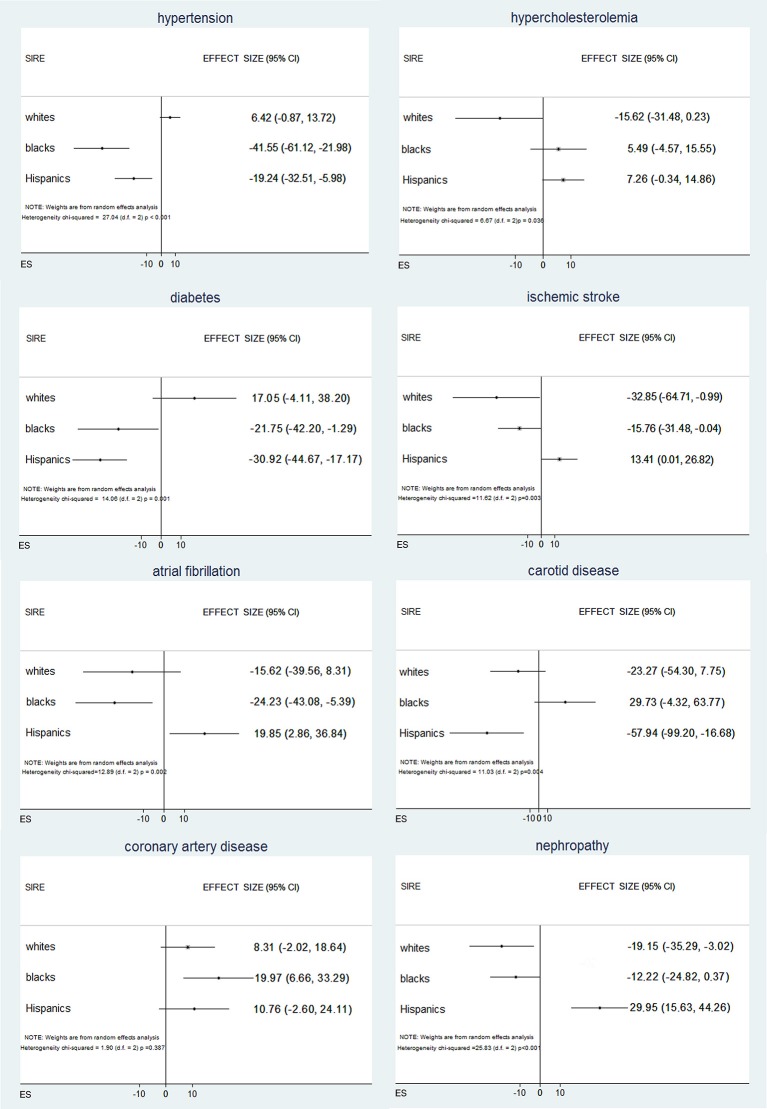
Associations between vascular risk factors and genetic ancestry across self-identified race/ethnicities within each self-identified race/ethnicities (SIRE) group, DNA ancestry is independently associated with vascular risk factor, with differences that vary across SIRE and vascular risk factor.

To further test these associations, we regressed VRF status against the AIM-derived PCs. Logistic regressions for VRFs adjusted for age, sex, and SIRE identified significant independent associations of genetic ancestry with all the VRFs studied except hypercholesterolemia (Supplement Table VI). Inverse variance weighted random effects meta-analysis across SIRE indicated that different PCs were predictive of different VRFs (Supplement Table VII), suggesting that different ancestral subpopulations are enriched for different exposures. In this meta-analysis (Figure 2), risks for hypertension, diabetes, and atrial fibrillation varied significantly among self-reported blacks and Hispanics, when genetic ancestry was taken into account. Similarly, black and Hispanic individuals showed differing risks for ischemic stroke, while genetic ancestry modified risk for nephropathy only within the self-reported white population. Finally, based on ancestry, risk for carotid disease and coronary arthery disease was different in Hispanics and blacks, respectively. Comparable results were obtained when analyzing admixture-based AF and PC-derived ancestry (Supplement Tables VIII, IX).

### AIMs modestly improve ICH risk prediction over SIRE

To estimate the changes in the explanatory ability of prediction models that include AIM-derived PCs, two regression models for ICH risk were computed: the first with age, sex, hypertension, and SIRE (model 1), the second adding AIM-derived PCs to the covariates (model 2). Akaike information criterion (AIC) (6,294 for model 1 VS 6,286 for model 2) and likelihood ratio test (*p* < 0.001) demonstrate superiority of the AIM-inclusive model 2 over model 1. Results showed the same pattern when models were also computed with centers of recruitments and smoking use, as covariates (AIC 6203.9 for model without ancestry VS 6185.0 for model with ancestry).

Our NRI analysis revealed similar results. Reclassifications for subjects with and without ICH events are summarized in Supplement Table X. For 57 subjects who did not experience ICH, classification improved using the model with AIMs, and for nine subjects it became worse. The NRI was estimated at 0.013 (0.004–0.021; *p* = 0.006), reflecting that individuals with ICH were 1.3% more likely to move up a category than down, compared with individuals without ICH.

## Discussion

In this multi-ethnic study of ICH, SIRE did not fully capture relevant genetic ancestry. Inclusion of AIMs substantially improved modeling of risk factor exposures. While inclusion of AIMs in an ICH prediction model improved model fit over SIRE alone, the overall improvement was modest. Certainly, deploying this approach in different racial and ethnic populations, as well as alternative risk exposures and disease states, are likely to reveal situations in which genetic ancestral modeling results in more dramatic improvements in risk prediction.

Social and cultural aspects such as access or utilization of care undoubtedly affect risk and outcome of disease. SIRE may capture exposures that vary by social background more closely than AIMs alone, and as such there would not appear to be any reason to fully replace self-reported data. However, a better understanding of genetic ancestry could help to understand why racial/ethnic minority groups have a lower risk for short-term all-cause mortality after being hospitalized for ischemic stroke than non-Hispanic whites, or why stratification by race does not improve risk prediction in high-risk hypertensive patients (32, 33). Such differences could be due to biological variation, but could also reflect differences in exposures that vary socioculturally within racial and ethnic groups in ways that are not fully captured by SIRE.

Rather than focusing on the magnitude of improvement with the incorporation of genetic ancestry into ICH prediction models, we submit that our results may serve as a proof of concept of for our approach to racial and ethnic modeling. Our analysis leveraged a limited pool of AIMs. It is likely that as the number of genetic markers increases, the fitness of the model will also improve (34). Further, given the high degree of stratification of VRFs by genetic ancestry in our analysis, we expect that other conditions for which highly stratified risk factors explain an even greater proportion of overall disease risk variance will demonstrate a greater improvement in modeling through inclusion of AIMs. For ICH, changes in AIC were small but reflect the inherent challenges in risk prediction in complex human disease irrespective of racial/ethnic contributions.

Adjusting for population stratification using AIMs improves statistical power in genetic association studies, but less information is available on whether these benefits extend to non-genetic studies (35). There are conflicting reports regarding the ability of SIRE to serve as an accurate predictor of population clusters (36–39). Prior studies have failed to demonstrate a greater predictive power of the genetic based ancestry over the SIRE-based categorization for cardiovascular disease (CVD) risk factors (40, 41). However, in these studies, AIMs were used to construct a continuous PC distribution that was then collapsed into to a categorical variable for analysis. Given the substantial overlap between ancestral population clusters in our study, our approach of using AIM-derived PCs as a continuous variable throughout our analyses may account for the differences in results.

Consistent with prior studies, we found substantial evidence of genetic clustering among the self-identified racial and ethnic populations studied (13). Also, as seen in other studies, the admixed nature of Hispanic populations results in a less compact (37) cluster than white or black populations. In our study, self-identified race generally corresponded well with genetic population clusters, but did not fully overlap. This correspondence is dependent on both the homogeneity within groups as well the sample size. Therefore, cluster analyses based on reported ancestry alone may overlook important components of population structure not captured by SIRE classification.

Beyond our confirmation of previous findings regarding genetic variance captured by SIRE (13) and the improvement of AIMs in defining populations (42), our results demonstrate the promising approach of adding genetic ancestry to the study of epidemiologic risk factors. Ascertainment of a small collection of DNA variants similar to those employed herein could be easily accomplished from blood or saliva in a small direct genotyping assay. Even if the residual benefit to including genetic ancestry remains modest, opmizing patient risk assessment by ancestry may still represent an important step forward in improving health equity as it pertains to providing maximally useful precision risk estimation across representative populations (43).

Strengths of this study include its large sample size, with a geographically diverse population well powered across representative race and ethnic populations. ICH cases and controls were well-characterized, with thorough clinical phenotyping of VRFs and centralized genotyping. Also, our results were confirmed by two distinctive and orthogonal methods for estimating ancestry. Several limitations of our approach deserve mention. As noted, ICH risk model improvements were modest after inclusion of genetic ancestry information. Second, VRF diagnoses were based on subject interview and could be inaccurate, although they were cross-validated and highly structured. Third, our analyses focused on non-Hispanic white, non-Hispanic black, and Hispanic populations. While these racial and ethnic groups are highly prevalent in the US, and are often included in studies of risk factors in US populations, we cannot extrapolate our findings to studies of other racial groups, or in focused studies of highly stratified subpopulations. Future studies in other cardiometabolic traits will be needed to support the generalizability of our approach.

In summary, genetically-defined ancestry provides additional risk exposure information that is not captured by SIRE alone. Inclusion of AIMs reveals additional stratification among risk factors between and within whites, blacks, and Hispanics. While differences in final ICH model specificity are small, incorporation of genetic ancestry into risk models may be superior in highly heterogeneous clinical poulations where risk exposures are highly correlated with race and ethnicity. Additional studies across diverse ancestries and risk exposures are needed to confirm and extend these findings.

## Ethics statement

This study was carried out in accordance with the recommendations of the Ethical Principles and Guidelines for the Protection of Human Subjects of Research, generally known as the Belmont Report, Partners Human Research Committee. The protocol was approved by the Partners Institutional Review Boards (IRBs).

## Author contributions

SM study concept and design, analysis and interpretation of data, drafting of the manuscript. UL and KC analysis and interpretation of data, drafting of the manuscript. CM acquisition of data, analysis and interpretation of data. FT, SK, MJ, and DW critical revision of manuscript for intellectual content, study supervision. CL critical revision of manuscript for intellectual content, acquisition of data, study design, analysis and interpretation of data, study supervision. JR study concept and design, critical revision of manuscript for intellectual content, drafting of the manuscript, study supervision. CA study concept and design, drafting of the manuscript, analysis and interpretation of data, study supervision.

### Conflict of interest statement

The authors declare that the research was conducted in the absence of any commercial or financial relationships that could be construed as a potential conflict of interest.
