# Supplementary material for: Comparison of Genetic and Self-Identified Ancestry in Modeling Intracerebral Hemorrhage Risk
Source: Front Neurol. 2018 Jul 6;9:514. doi: 10.3389/fneur.2018.00514 (PMC6043667; doi:10.3389/fneur.2018.00514)

**ONLINE SUPPLEMENT**

**Comparison of genetic and self-identified ancestry in modeling intracerebral hemorrhage risk**

Sandro Marini ^1,2^, MD; Umme K. Lena ^1,2^, BA; Katherine M. Crawford ^1,2^, BS;

Charles J. Moomaw ^3^, PhD; Fernando D. Testai ^4^, MD; Steven J. Kittner ^5^, MD, MPH; Michael L. James ^6^, MD, FAHA; Daniel Woo ^3^, MD; Carl D. Langefeld ^7^, PhD;

Jonathan Rosand ^1,2^, MD, MSc; Christopher D. Anderson ^1,2^, MD, MMSc;

on behalf of the ERICH investigators

1) Center for Genomic Medicine, Massachusetts General Hospital, Boston, MA, USA

2) Program in Medical and Population Genetics, Broad Institute, Cambridge, MA, USA

3) Department of Neurology and Rehabilitation Medicine, University of Cincinnati College of Medicine, Cincinnati, OH, USA

4) Department of Neurology and Rehabilitation, University of Illinois College of Medicine, Chicago, IL, US

5) Department of Neurology, Baltimore Veterans Administration Medical Center and University of Maryland School of Medicine, Baltimore, MD, USA

6) Brain Injury Translational Research Center, Departments of Anesthesiology and Neurology, Duke University, Durham, NC, USA

7) Center for Public Health Genomics and Department of Biostatistical Sciences, Wake Forest University, Winston-Salem, NC, USA

**Supplement** **Table I:** Ancestry informative markers selected

| AIM | Chromosome | position (GRCh37.p13) | A1 | A2 | Allele frequencies | | |
| --- | --- | --- | --- | --- | --- | --- | --- |
|  | | | | | AFR | AMR | EUR |
| rs7581299 | 2 | 179900720 | T | C | 0.749 | 0.660 | 0.370 |
| rs11725412 | 4 | 38277754 | A | G | 0.215 | 0.464 | 0.063 |
| rs12640848 | 4 | 71506412 | A | G | 0.967 | 0.660 | 0.331 |
| rs1423099 | 5 | 75427518 | T | C | 0.930 | 0.568 | 0.264 |
| rs4463276 | 6 | 145055331 | A | G | 0.088 | 0.376 | 0.771 |
| rs13259288 | 8 | 4483059 | T | G | 0.822 | 0.478 | 0.764 |
| rs12679427 | 8 | 72242674 | T | C | 0.437 | 0.424 | 0.724 |
| rs10962599 | 9 | 16795286 | T | C | 0.977 | 0.651 | 0.274 |
| rs10840311 | 11 | 9854857 | T | C | 0.726 | 0.601 | 0.352 |
| rs3019657 | 11 | 134511647 | A | G | 0.696 | 0.496 | 0.871 |
| rs550338 | 12 | 24512037 | A | G | 0.697 | 0.460 | 0.764 |
| rs200354 | 14 | 99375321 | T | G | 0.336 | 0.476 | 0.829 |
| rs12913832 | 15 | 28365618 | A | G | 0.972 | 0.798 | 0.364 |
| rs2216594 | 19 | 33533258 | A | G | 0.048 | 0.347 | 0.676 |
| rs801712 | 22 | 47090243 | C | G | 0.251 | 0.477 | 0.795 |

AIM: ancestry informative marker; A1 and A2: allele frequency; AFR: Afro-American; AMR: Mixed American; EUR: European

**Supplement** **Table II:** Percent of individuals reclassified based on ancestry informative marker (AIM) derived principal components (PC) versus self-identified race/ethnicity (SIRE).

| **Percent** **Reclassification based on AIM-derived PC** | | | | |
| --- | --- | --- | --- | --- |
|  |  | Hispanics | Hispanics or blacks | blacks |
| **S**  **I**  **R**  **E** | whites | 42 (2.4%) | 28 (1.6%) | 1 (0.1%) |
|  |  | | | |
|  |  | Hispanics | whites | Hispanics or whites |
|  | blacks | 21 (1.2%) | 5 (0.3%) | 18 (1.1%) |
|  |  | | | |
|  |  | blacks | whites |  |
|  | Hispanics | 24 (1.6 %) | 20 (1.3 %) |  |

Cluster identity and reclassification are based on proximity to the 95% data of each cluster in PCs space. (See also Supplemental figure 1). PCA: principal component analysis. AIM: ancestry informative markers. SIRE: self-identified race/ethnicity

**Supplement Table III:** correlation between ancestry fraction (AF 1- 3) obtained from ADMITURE and Principal Components (PC 1-3)

| **Pearson's ρ correlation** | | | | | | |
| --- | --- | --- | --- | --- | --- | --- |
|  | AF 1 | AF 2 | AF 3 | PC 1 | PC 2 | PC 3 |
| AF 1 | 1 | -0.28 | -0.46 | -0.57 | -0.79 | 0 |
| AF 2 | -0.28 | 1 | -0.72 | -0.62 | 0.76 | 0.02 |
| AF 3 | -0.46 | -0.72 | 1 | 0.99 | -0.13 | -0.02 |
| PC 1 | -0.57 | -0.62 | 0.99 | 1 | 0 | 0 |
| PC 2 | -0.79 | 0.76 | -0.13 | 0 | 1 | 0 |
| PC 3 | 0 | 0.02 | -0.02 | 0 | 0 | 1 |
| **p value** | | | | | | |
|  | AF 1 | AF 2 | AF 3 | PC 1 | PC 2 | PC 3 |
| AF 1 |  | <0.001 | <0.001 | <0.001 | <0.001 | 0.907 |
| AF 2 | <0.001 |  | <0.001 | <0.001 | <0.001 | 0.1038 |
| AF3 | <0.001 | <0.001 |  | <0.001 | <0.001 | 0.1562 |
| PC 1 | <0.001 | <0.001 | <0.001 |  | 0.947 | 0.8149 |
| PC 2 | <0.001 | <0.001 | <0.001 | 0.947 |  | 0.9951 |
| PC 3 | 0.907 | 0.1038 | 0.1562 | 0.8149 | 0.9951 |  |

**Supplement** **Table IV**: Distributions of ancestry fractions among cases and controls for each VRFs diagnosis. Similarly, to Figure 2, distribution of the diseases among SIRE is also reported. Among the three ancestry fractions we reported more significant one.

|  | **Hypertension** | | | | **p value** |
| --- | --- | --- | --- | --- | --- |
|  | **Yes** | | **No** | |  |
| **AF 3 Median (IQR)** | 0.291  (0.109-0.660) | | 0.432  (0.179 – 0.711) | | <0.001 |
|  | **Affected by hypertension** | | | |  |
|  | **blacks** | **whites** | | **Hispanics** |  |
| **n (%)** | 1269(74.8) | 1068(61.9) | | 969(65.8) | <0.001 |
|  | | | | | |
|  | **Hypercholesterolemia** | | | | **p value** |
|  | **Yes** | | **No** | |  |
| **AF 1 Median (IQR)** | 0.177  (0.021 – 0.345) | | 0.207  (0.076 – 0.378) | | <0.001 |
|  | **Affected by hypercholesterolemia** | | | |  |
|  | **blacks** | **whites** | | **Hispanics** |  |
| **n (%)** | 840(49.9) | 614(37.0) | | 588(41.3) | <0.001 |
|  | | | | | |
|  | **Diabetes** | | | | **p value** |
|  | **Yes** | | **No** | |  |
| **AF 1 Median (IQR)** | 0.239  (0.068 – 0.410) | | 0.187  (0.045 – 0.351) | | <0.001 |
|  | **Affected by diabetes** | | | |  |
|  | **Blacks** | **whites** | | **Hispanics** |  |
| **n (%)** | 318(18.4) | 386(22.7) | | 436(29.4) | <0.001 |
|  | | | | | |
|  | **Coronary artery disease** | | | | **p value** |
|  | **Yes** | | **No** | |  |
| **AF 1 Median (IQR)** | 0.160  (0.001 – 0.321) | | 0.199  (0.055 – 0.367) | | <0.001 |
|  | **Affected by coronary artery disease** | | | |  |
|  | **blacks** | **whites** | | **Hispanics** |  |
| **n (%)** | 120(7.1) | 245(14.2) | | 127(8.6) | <0.001 |
|  | | | | | |
|  | **Atrial fibrillation** | | | | **p value** |
|  | **Yes** | | **No** | |  |
| **AF 1 Median (IQR)** | 0.112  (0.001 – 0.267) | | 0.201  (0.061 – 0.372) | | <0.001 |
|  | **Affected by atrial fibrillation** | | | |  |
|  | **Blacks** | **whites** | | **Hispanics** |  |
| **n (%)** | 64(3.8) | 214(12.4) | | 66(4.4) | <0.001 |
|  | | | | | |
|  | **History of ischemic stroke** | | | | **p value** |
|  | **Yes** | | **No** | |  |
| **AF 3 Median (IQR)** | 0.294  (0.090 - 0.634) | | 0.336  (0.128 – 0.685) | | 0.045 |
|  | **History of ischemic stroke** | | | |  |
|  | **Blacks** | **whites** | | **Hispanics** |  |
| **n (%)** | 97 (5.8) | 94 (5.4) | | 99 (6.7) | 0.312 |
|  | | | | | |
|  | **History of nephropathy** | | | | **p value** |
|  | **Yes** | | **No** | |  |
| **AF 1 Median (IQR)** | 0.248  (0.078-0.418) | | 0.192  (0.047 – 0.359) | | 0.003 |
|  | **History of nephropathy** | | | |  |
|  | **blacks** | **whites** | | **Hispanics** |  |
| **n (%)** | 127(7.5) | 84(4.9) | | 14(7.0) | 0.003 |
|  | | | | | |
|  | **History of carotid disease** | | | | **p value** |
|  | **Yes** | | **No** | |  |
| **AF 2 Median (IQR)** | 0.233  (0.110 – 0.376) | | 0.318  (0.145 – 0.588) | | <0.001 |
|  | **History of carotid disease** | | | |  |
|  | **blacks** | **whites** | | **Hispanics** |  |
| **n (%)** | 17(1.0) | 54(3.1) | | 18(1.2) | <0.001 |

AF: ancestry fraction; SIRE: self-identified race/ethnicity; VRFs: vascular sick factor

**Supplement** **Table V**: Distributions of ancestry fractions among cases and controls for VRFs diagnosis within each self-identified race/ethnicity (Ancestry fraction with the lowest p value is shown).

|  | **Hypertension** | | | **Standardized Test Statistic** | **p value** |
| --- | --- | --- | --- | --- | --- |
|  | **AF** | **Yes** | **No** |  |  |
| **whites** | 1 | MR= 837.29 | MR= 865.34 | -1.189 | 0.234 |
| **blacks** | 3 | MR= 815.24 | MR= 900.09 | -3.141 | 0.002 |
| **Hispanics** | 2 | MR= 716.20 | MR= 744.80 | -1.234 | 0.217 |
|  | | | | | |
|  | **Hypercholesterolemia** | | |  |  |
|  |  | **Yes** | **No** |  |  |
| **whites** | 1 | MR= 837.29 | MR= 865.34 | -1.189 | 0.234 |
| **blacks** | 3 | MR= 815.24 | MR= 900.09 | -3.141 | 0.002 |
| **Hispanics** | 2 | MR= 716.20 | MR= 744.80 | -1.234 | 0.217 |
|  | | | | | |
|  | **Diabetes** | | |  |  |
|  | **AF** | **Yes** | **No** |  |  |
| **whites** | 1 | MR= 816.12 | MR= 858.87 | -1.443 | 0.149 |
| **blacks** | 3 | MR= 797.76 | MR= 851.85 | -1.936 | 0.053 |
| **Hispanics** | 3 | MR= 678.14 | MR= 754.72 | -2.545 | 0.002 |
|  | | | | | |
|  | **Coronary artery disease** | | |  |  |
|  | **AF** | **Yes** | **No** |  |  |
| **whites** | 3 | MR= 813.58 | MR= 851.36 | -1.110 | 0.267 |
| **blacks** | 2 | MR=753.48 | MR= 842.33 | -1.953 | 0.051 |
| **Hispanics** | 3 | MR= 821.73 | MR=720.22 | 2.588 | 0.010 |
|  | | | | | |
|  | **Atrial fibrillation** | | |  |  |
|  | **AF** | **Yes** | **No** |  |  |
| **whites** | 3 | MR= 722.51 | MR= 857.38 | -2.578 | 0.010 |
| **blacks** | 2 | MR= 881.85 | MR= 826.28 | 1.104 | 0.270 |
| **Hispanics** | 3 | MR= 680.21 | MR= 732.48 | -1.182 | 0.237 |
|  | | | | | |
|  | **History of ischemic stroke** | | |  |  |
|  | **AF** |  |  |  |  |
| **whites** | 3 | MR= 722.51 | MR= 857.38 | -2.578 | 0.010 |
| **blacks** | 2 | MR= 881.85 | MR= 826.28 | 1.104 | 0.270 |
| **Hispanics** | 3 | MR= 680.21 | MR= 732.48 | -1.182 | 0.237 |
|  | | | | | |
|  | **History of nephropathy** | | |  |  |
|  | **AF** | **Yes** | **No** |  |  |
| **whites** | 1 | MR=792.21 | MR=851.36 | -1.107 | 0.268 |
| **blacks** | 1 | MR= 878.52 | MR= 834.16 | 0.991 | 0.321 |
| **Hispanics** | 1 | MR=824.60 | MR= 721.65 | 2.405 | 0.016 |
|  | | | | | |
|  | **History of carotid disease** | | |  |  |
|  | **AF** | **Yes** | **No** |  |  |
| **whites** | 1 | MR= 905.06 | MR= 846.64 | 0.864 | 0.388 |
| **blacks** | 1 | MR= 963.35 | MR= 838.23 | 1.060 | 0.289 |
| **Hispanics** | 2 | MR= 734.12 | MR= 521.11 | -2.129 | 0.033 |

AF: ancestry fraction MR: mean rank; VRF: vascular risk factor

**Supplement** **Table VI:** Regression of PCs on VRF (adjusted for age, sex and SIRE)

|  | **beta** | **S.E** | **p value** |
| --- | --- | --- | --- |
|  | **Hypertension** | | |
| **PC1** | -14.159 | 4.399 | 0.001 |
|  | **Diabetes** | | |
| **PC1** | -16.034 | 4.699 | 0.001 |
|  | **Hypercholesterolemia** | | |
| **PC3** | 3.773 | 2.143 | 0.078 |
|  | **History of atrial fibrillation** | | |
| **PC8** | 8.810 | 4.346 | 0.043 |
|  | **History of coronary artery disease** | | |
| **PC3** | 9.431 | 3.489 | 0.007 |
|  | **History of ischemic stroke** | | |
| **PC1** | -28.735 | 8.443 | 0.001 |
|  | **History of nephropathy** | | |
| **PC5** | 12.708 | 4.099 | 0.002 |
|  | **History of carotid disease** | | |
| **PC10** | -26.951 | 7.747 | 0.001 |

VRFs: vascular risk factors; PCs: principal components; SIRE: self-identified race and ethnicity; SE: standard error

**Supplement** **Table VII:** PCs selected by backward stepwise regression (likelihood ratio) for each VRFs within each SIRE

|  | **Hypertension** | | | **Diabetes** | | |
| --- | --- | --- | --- | --- | --- | --- |
| SIRE | whites | blacks | Hispanics | whites | blacks | Hispanics |
| Best PC selected | 3 | 1 | 1 | 1 | 1 | 1 |
| beta | 6.422 | -41.547 | -19.243 | 17.046 | -21.745 | -30.918 |
| S.E. | 3.721 | 9.984 | 6.767 | 10.793 | 10.434 | 7.016 |
| p value | 0.084 | <0.001 | 0.04 | 0.114 | 0.037 | <0.001 |
|  | **History of atrial fibrillation** | | | **History of coronary artery disease** | | |
| SIRE | whites | blacks | Hispanics | whites | blacks | Hispanics |
| Best PC selected | 1 | 4 | 3 | 3 | 3 | 5 |
| beta | -15.624 | -24.233 | 19.849 | 8.314 | 19.972 | 10.755 |
| S.E. | 12.212 | 9.615 | 8.670 | 5.270 | 6.793 | 6.814 |
| p value | 0.201 | 0.012 | 0.022 | 0.115 | 0.003 | 0.114 |
|  | **Hypercholesterolemia** | | | **History of ischemic stroke** | | |
| SIRE | whites | blacks | Hispanics | whites | blacks | Hispanics |
| Best PC selected | 1 | 2 | 5 | 1 | 5 | 4 |
| beta | -15.624 | 5.488 | 7.264 | -32.849 | -15.760 | 13.414 |
| S.E. | 8.089 | 5.134 | 3.878 | 16.256 | 8.023 | 6.839 |
| p value | 0.053 | 0.285 | 0.061 | 0.043 | 0.049 | 0.050 |
|  | **History of nephropathy** | | | **History of carotid disease** | | |
| SIRE | whites | blacks | Hispanics | whites | blacks | Hispanics |
| Best PC selected | 6 | 8 | 5 | 8 | 7 | 2 |
| beta | -19.154 | -12.222 | 29.946 | -23.274 | 29.725 | -57.939 |
| S.E. | 8.234 | 6.427 | 7.305 | 15.831 | 17.372 | 21.053 |
| p value | 0.020 | 0.057 | <0.001 | 0.142 | 0.087 | 0.006 |

VRFs: vascular risk factors; PCs: principal components; SIRE: self-identified race and ethnicity

**Supplement** **Table VIII:** Regression of Ancestry fraction on VRF (adjusted for age, sex and SIRE)

|  | **beta** | **S.E** | **p value** |
| --- | --- | --- | --- |
| **Hypertension** | | | |
| **AF 1** | 2.19 | 1.36-3.53 | < 0.001 |
| **Diabetes** | | | |
| **AF 1** | 2.44 | 1.47 – 4.03 | < 0.001 |
| **Hypercholesterolemia** | | | |
| **AF 2** | 1.54 | 0.99 - 2.40 | 0.057 |
| **History of atrial fibrillation** | | | |
| **AF 1** | 0.67 | 0.26 – 1.71 | 0.396 |
| **History of coronary artery disease** | | | |
| **AF 2** | 1.28 | 0.60 – 2.71 | 0.527 |
| **History of ischemic stroke** | | | |
| **AF 2** | 3.79 | 1.53 - 9.41 | 0.004 |
| **History of nephropathy** | | | |
| **AF 1** | 2.11 | 0.89 – 4.99 | 0.088 |
| **History of carotid disease** | | | |
| **AF 1** | 2.13 | 0.39 - 11.624 | 0.381 |

VRFs: vascular risk factors; AF: ancestry fraction; SIRE: self-identified race and ethnicity; SE: standard error

**Supplement** **Table IX:** Ancestry fraction selected by backward stepwise regression (likelihood ratio) for each VRFs within each SIRE

|  | **Hypertension** | | | **Diabetes** | | |
| --- | --- | --- | --- | --- | --- | --- |
| SIRE | whites | blacks | Hispanics | whites | blacks | Hispanics |
| Best AF selected | 1 | 1 | 1 | 1 | 2 | 1 |
| beta | -0.524 | 2.28 | 0.986 | -1.05 | 1.25 | 1.40 |
| S.E. | 0.52 | 0.59 | 0.33 | 0.69 | 0.52 | 0.34 |
| p value | 0.312 | <0.001 | 0.003 | 0.128 | 0.016 | <0.001 |
|  | **History of atrial fibrillation** | | | **History of coronary artery disease** | | |
| SIRE | whites | blacks | Hispanics | whites | blacks | Hispanics |
| Best AF selected | 1 | 1 | 1 | 2 | 1 | 2 |
| beta | 0.71 | -2.28 | -0.34 | 0.49 | 2.66 | -0.38 |
| S.E. | 0.58 | 1.23 | 0.70 | 0.56 | 1.01 | 0.53 |
| p value | 0.228 | 0.064 | 0.624 | 0.385 | 0.008 | 0.47 |
|  | **Hypercholesterolemia** | | | **History of ischemic stroke** | | |
| SIRE | whites | blacks | Hispanics | whites | blacks | Hispanics |
| Best AF selected | 2 | 2 | 2 | 2 | 2 | 1 |
| beta | 1.06 | 0.21 | 0.08 | 2.22 | 1.522 | 1.08 |
| S.E. | 0.40 | 0.45 | 0.37 | 0.76 | 0.95 | 0.61 |
| p value | 0.008 | 0.65 | 0.83 | 0.004 | 0.110 | 0.075 |
|  | **History of nephropathy** | | | **History of carotid disease** | | |
| SIRE | whites | blacks | Hispanics | whites | blacks | Hispanics |
| Best AF selected | 1 | 1 | 1 | 1 | 1 | 2 |
| beta | -1.473 | 0.94 | 0.95 | -0.48 | 3.18 | -2.42 |
| S.E. | 1.325 | 0.95 | 0.58 | 1.56 | 2.65 | 1.79 |
| p value | 0.266 | 0.322 | 0.099 | 0.759 | 0.231 | 0.175 |

VRFs: vascular risk factors; AF: ancestry fraction; SIRE: self-identified race and ethnicity

**Supplement** **Table X:** Reclassification among patients with and without ICH given by the two models with and without genetic factors

|  | Model without  AIM-derived PCs | Model with  AIM-derived PCs | | Net correctly reclassified (%) |
| --- | --- | --- | --- | --- |
|  |  | < 30% | >31 % |  |
| Subject without ICH | $\leq$ 30% | 16 | 9 | 2.2 |
|  | >31 % | 57 | 2025 |  |
| Subject with ICH | $\leq$ 30% | 15 | 8 | 1.0 |
|  | >31 % | 36 | 2652 |  |

ICH: intracerebral hemorrhage; PC: principal components: AIM: ancestry informative markers

**Supplement** **Figure I:** Dimensional plot of principal components grouped by self-identified race/ancestry (SIRE) with ellipses encompassing the 95 % clusters of data

**
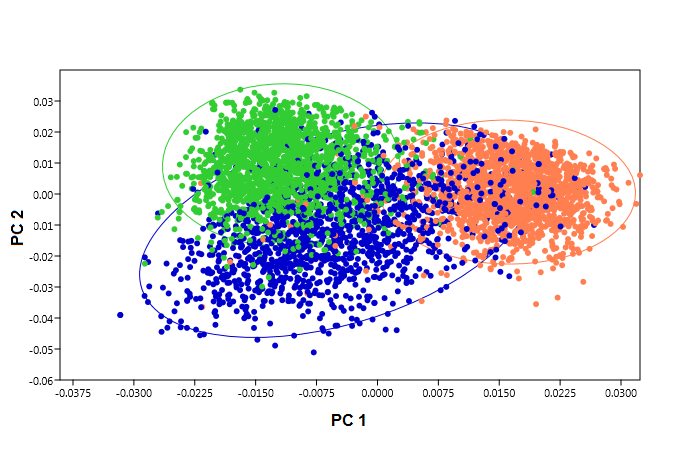
**

**Supplement** **Figure II:** Scatterplot matrix of the first 7 principal components.**
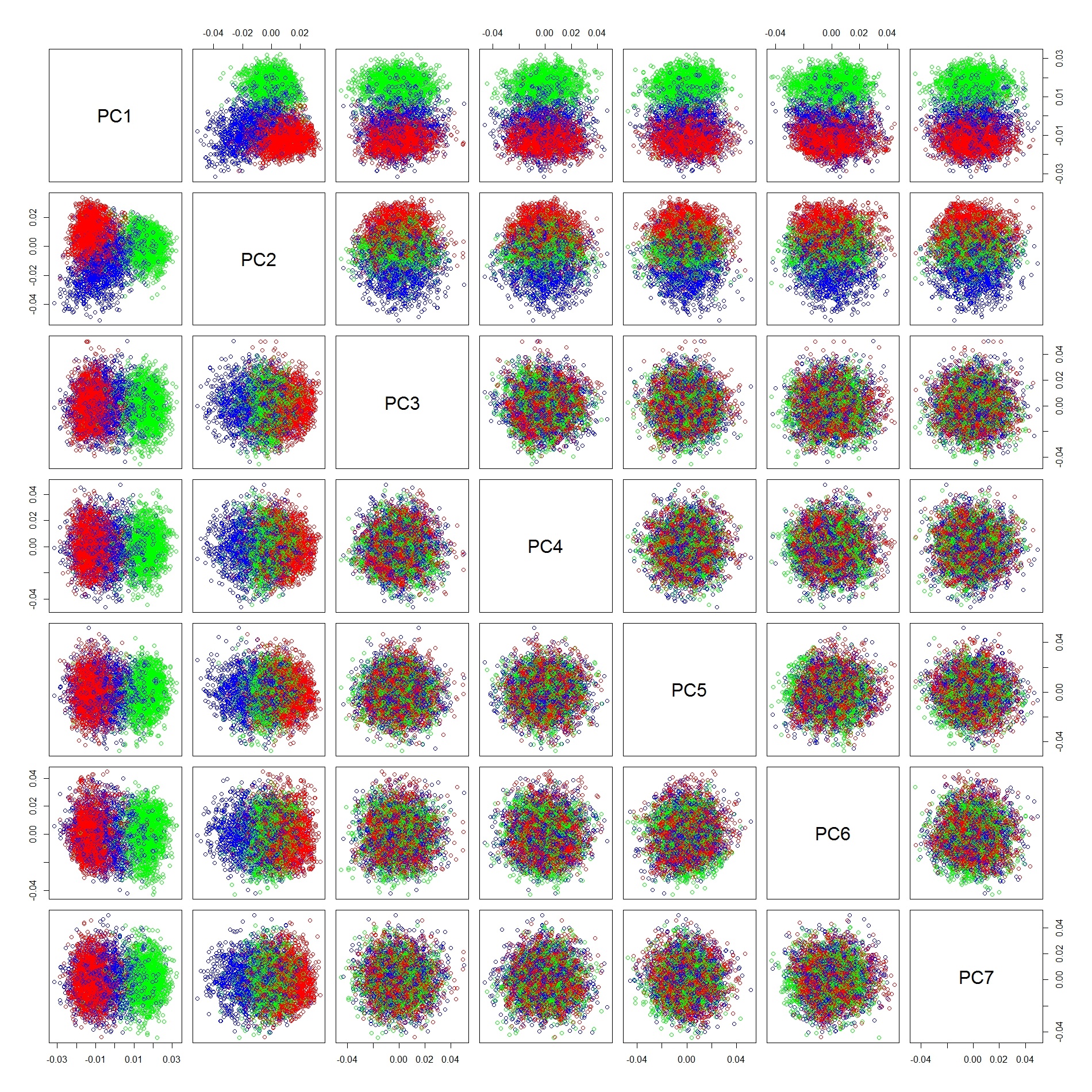
**

**Supplement** **Figure III:** bar plot representing the three Ancestry Faction computed from ADMIXTURE for each subject, grouped by their self-identified race/ethnicity

**
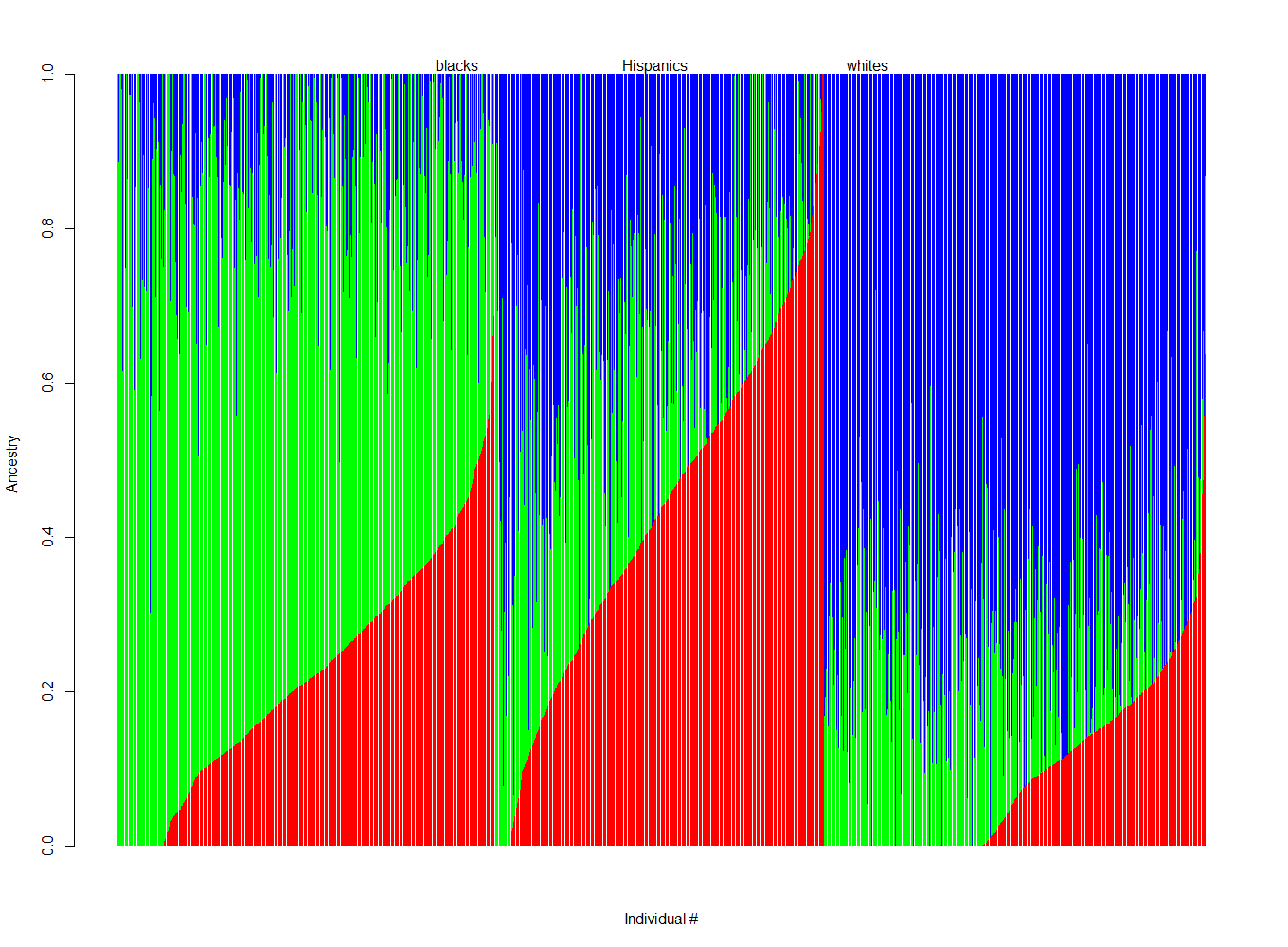
**

**Supplement** **Figure IV:** Mann-Whitney U Test distributions demonstrating that PC1 differentiates individuals with and without hypertension, diabetes, CAD, nephropathy, ischemic stroke and atrial fibrillation.


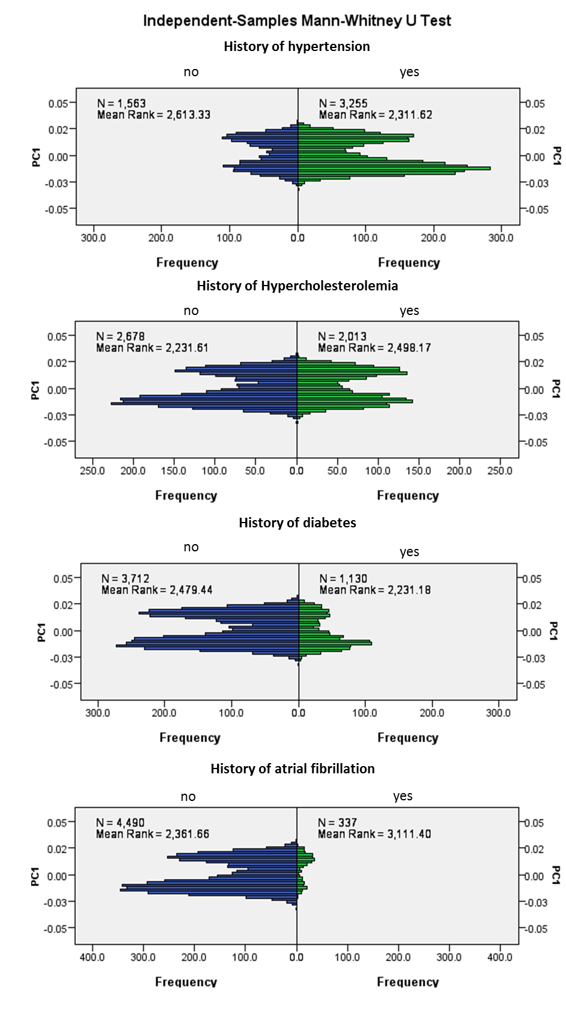


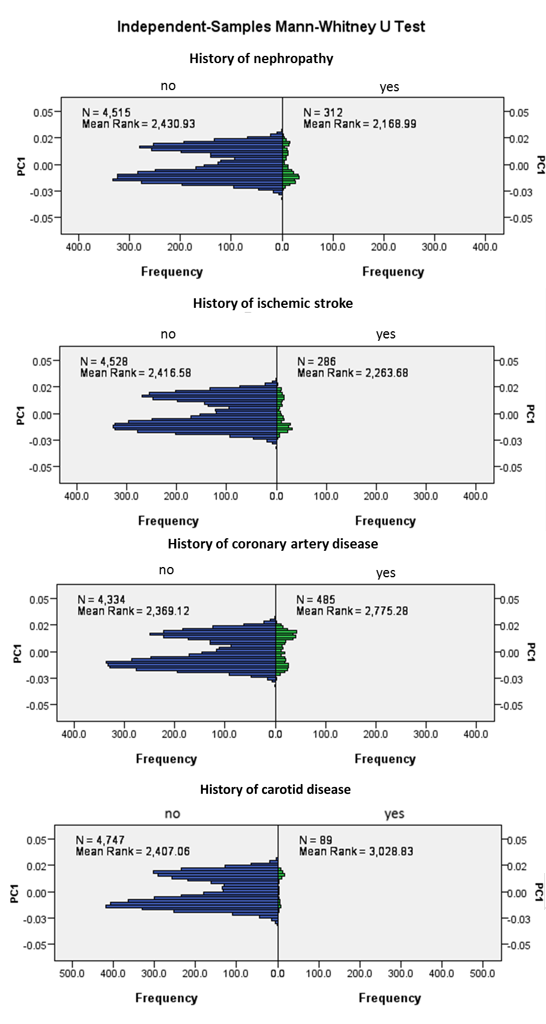

Supplement: Supplementary file 1 [file Data_Sheet_1.docx]
